# Transposition der A. mesenterica superior an der infrarenalen Aorta beim „Nussknacker“-Syndrom

**DOI:** 10.1055/a-2636-2267

**Published:** 2025-07-17

**Authors:** Stephan Arndt, Frank Meyer, Udo Barth, Maciej Pech, Zuhir Halloul

**Affiliations:** 1 Arbeitsbereich Gefäßchirurgie; Klinik für Allgemein-, Viszeral-, Gefäß- und Transplantationschirurgie, Otto-von-Guericke-Universität mit Universitätsklinikum, Magdeburg, Deutschland; 2 Klinik für Allgemein-, Viszeral-, Gefäß- und Transplantationschirurgie, Otto-von-Guericke-Universität mit Universitätsklinikum, Magdeburg, Deutschland; 3 Klinik für Radiologie und Nuklearmedizin, Otto-von-Guericke-Universität mit Universitätsklinikum, Magdeburg, Deutschland

**Keywords:** Nussknacker-Syndrom, May-Turner-Syndrom, Wilkie-Syndrom, aorto-mesenterialer Winkel, Schnabelzeichen, „pelvic congestion syndrome“, nut cracker-syndrome, May-Turner syndrome, Wilkie syndrome, aorto-mesenteric angle, pelvic congestion syndrome

## Abstract

**Hintergrund:**

Das „Nussknacker“-Syndrom ist ein sicher unterdiagnostiziertes Krankheitsbild, mit dem die Patienten häufig einen längeren Leidensweg zurücklegen, ehe es adäquat diagnostiziert und behandelt wird. Hinsichtlich der Behandlung existiert eine Fülle unterschiedlicher therapeutischer Maßnahmen, wobei die am ehesten kausal anmutende Maßnahme, die Transposition der A. mesenterica superior, im Verhältnis wenig eingesetzt wird.

**Ziel:**

Basierend auf selektiven Referenzen der einschlägigen medizinisch-wissenschaftlichen Literatur und eigenen klinisch-gefäßmedizinischen Erfahrungen sollen die wesentlichen Charakteristika des „Nussknacker“-Syndroms dargestellt werden.

**Methode:**

Es wurde ein narrativer Review unter Literatursuche in PubMed unter Nutzung der Stichworte „Nussknacker-Syndrom“, „May-Turner-Syndrom“, „Wilkie-Syndrom“, „aortomesenterialer Winkel“, „pelvic congestion syndrome“ zum Thema verfasst.

**Ergebnisse:**

Die Beschwerden des Nussknacker-Syndroms sind unspezifisch und mannigfaltig und sollten gerade in ihrer Kombination an ein Nussknacker-Syndrom denken lassen. Duplexsonografie und CT sind Methoden hoher diagnostischer Genauigkeit und können zudem relativ häufig simultan auftretende weitere Gefäßanomalien nachweisen. In Zusammenschau der Klinik und Paraklinik kann patientenspezifisch ein Therapieplan – a. e. im Rahmen eines Stufenkonzeptes – festgelegt werden, in dem auch die Transposition der A. mesenterica superior ein kausales, effektives und sicheres Verfahren gerade bei kombinierten Gefäßanomalien wie May-Turner- oder Wilkie-Syndrom darstellt.

**Schlussfolgerung:**

Das Nussknacker-Syndrom als venöse Abflussbehinderung der Niere stellte, medizinisch gesehen, bislang selbst eine recht „harte Nuss“ dar, wofür die moderne Medizin durch Evidenzfindung nicht zuletzt durch Fallberichte und retrospektive Kohortenanalysen mittlerweile einen verifizierten diagnostischen und therapeutischen Algorithmus bereithält.

## Abstract (en)

**Background:**

The nut cracker syndrome (NCS) can be considered as an under-diagnosed disease, which the patients have usually suffered from for a prolonged time prior to correct diagnosis and subsequent initiation of adequate therapy. There are several possible therapies, the most promising – transposition of the superior mesenteric artery (SMA) – from the perspective of its aetiopathogenesis – has rarely been used.

**Aim:**

Based on selective references from the scientific medical literature and our own clinical experiences in vascular medicine, the important characteristics of the “nut cracker” phenomenon and syndrome are described.

**Method:**

A topic-related narrative review has been based on a literature search in PubMed using the key words “Nut cracker syndrome”, “May-Turner syndrome“, “Wilkie syndrome”, “aorto-mesenteric angle“, “pelvic congestion syndrome”.

**Results:**

There are diverse symptoms in NCS, which is unspecific; however, in particular, the combination of signs and symptoms associated with a suspicious pathological urine finding may indicate NCS. Duplex ultrasonography and CT-scan are very accurate diagnostic procedures and include an adequate search for simultaneously occurring vascular anomalies. Taken together, clinical findings and laboratory parameters may lead to a patient-specific and graduated therapeutic plan, in which transposition of SMA is a causal, effective and safe procedure, particularly when there are combined vascular anomalies, e.g., the combination of NCS with May-Turner syndrome in young patients.

**Conclusion:**

The nutcracker syndrome as venous congestion of the kidney may be a “hard nut”. Modern medicine aims at achieving evidence for the best approach by case reports and retrospective cohort studies and can – in the meanwhile – provide a verified diagnostic work-up. In addition, is a therapeutic algorithm comprising a spectrum of highly effective measures, particularly for young patients, who frequently exhibit additional vascular anomalies.

## Einleitung

Das Nussknacker-Syndrom ist ein sicher unterdiagnostiziertes Krankheitsbild, mit dem die Patienten häufig einen längeren Leidensweg zurücklegen, ehe es adäquat diagnostiziert und behandelt wird. Hinsichtlich der Behandlung existiert eine Fülle unterschiedlicher therapeutischer Maßnahmen, wobei die am ehesten kausal anmutende Maßnahme, die Transposition der A. mesenterica superior, im Verhältnis wenig eingesetzt wird.

Das Ziel des Manuskripts war es, basierend auf selektiven Referenzen der einschlägigen medizinisch-wissenschaftlichen Literatur und eigenen klinisch-gefäßmedizinischen Erfahrungen die wesentlichen Charakteristika des „Nussknacker“-Phänomens und -Syndroms darzustellen.

## Methode

Es wurde ein narrativer Review unter Literatursuche in PubMed mit Nutzung der Stichworte „Nussknacker-Syndrom“, „May-Turner-Syndrom“, „Wilkie-Syndrom“, „aortomesenterialer Winkel“, „pelvic congestion syndrome“ zum Thema verfasst.

## Ergebnisse

### Definition

**Abb. 1:**
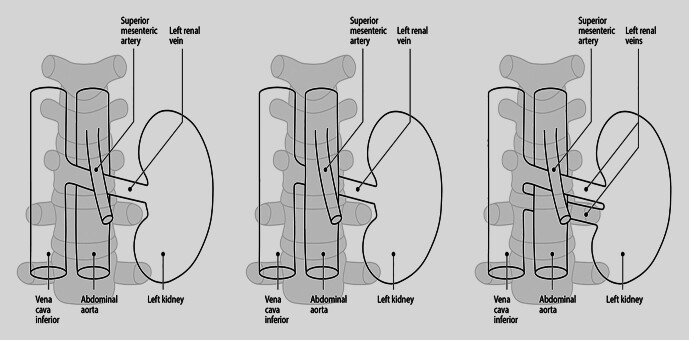
Schemata zu Anlagevarianten der V. renalis sinistra (1. Abbildung mit vorderem Nussknacker-Syndrom mit potenzieller Venenkompression zwischen A. mesenterica superior und Aorta abdominalis, 2. Abbildung mit hinterem Nussknacker-Syndrom mit potenzieller Venenkompression zwischen Aorta abdominalis und Wirbelsäule und 3. Abbildung mit kombiniertem Nussknacker-Syndrom – sog. „Circumaortic renal Collar“). Quelle: Image- & Bilderfundus des Arbeitsbereiches Gefäßchirurgie, Klinik für Allgemein-, Viszeral-, Gefäß- und Transplantationschirurgie Uniklinik Magdeburg.

Der Begriff Nussknacker-Syndrom, engl. „Nutcracker Syndrome“ (Abkürzung: NCS) wurde 1972 durch de Schepper 1 geprägt und beschreibt die symptomatische „nussknackerartige“ Einklemmung der V. renalis sinistra zwischen A. mesenterica superior und Aorta abdominalis ( Abb. 1 ). Aufgrund anatomischer Varianten der venösen Drainage der linken Niere stellt diese Konfiguration in fast 90% der Fälle die Regelvariante dar und wird als vorderes Nussknacker-Syndrom bezeichnet. In 1,5% der Fälle verläuft die linke Nierenvene dorsal der Aorta abdominalis und kann zwischen selbiger und der Lendenwirbelsäule komprimiert werden, was als hinteres Nussknacker-Syndrom Eingang in die medizinische Terminologie gefunden hat. In 10% der Fälle sind eine ventral und eine dorsal der Aorta abdominalis verlaufende linke Nierenvene, sog. „Circumaortic renal Collar“, zu beobachten, wobei das entsprechende Kompressionssyndrom als kombiniertes Nussknacker-Syndrom bezeichnet wird 2 .

Ein rechtsseitiges Nussknacker-Syndrom ist durch die Kompression der rechten Nierenvene bedingt, sei es durch den Uterus in der Schwangerschaft oder durch eine linksseitig angelegte V. cava inferior, die durch eine Persistenz der embryologisch angelegten linken suprakardinalen Vene und Rückbildung der rechten suprakardinalen Vene mit einer Prävalenz von 0,2% bis 0,5% auftreten kann 3 .

Obwohl der Begriff des Nussknacker-Syndroms Eingang in die entsprechenden Standardwerke der Gefäßchirurgie und Anatomie gefunden hat, ist die Inzidenz desselbigen unbekannt. Dies mag daran liegen, dass sowohl die diagnostischen Zeichen (verkleinerter aortomesenterialer Winkel, Schnabelzeichen, Druckgradient > 3) ebenfalls bei gesunden Menschen vorkommen können als auch die klinische Symptomatik betroffener Patienten sehr unspezifisch ist, sodass die Erkrankung wahrscheinlich unterdiagnostiziert sein dürfte 4 .

Die Erkrankung manifestiert sich zumeist zwischen dem 20. und 50. Lebensjahr 5 und weist entgegen früherer Annahme keine geschlechtsabhängige Prävalenz auf 6 .

### Pathophysiologie

**Abb. 2:**
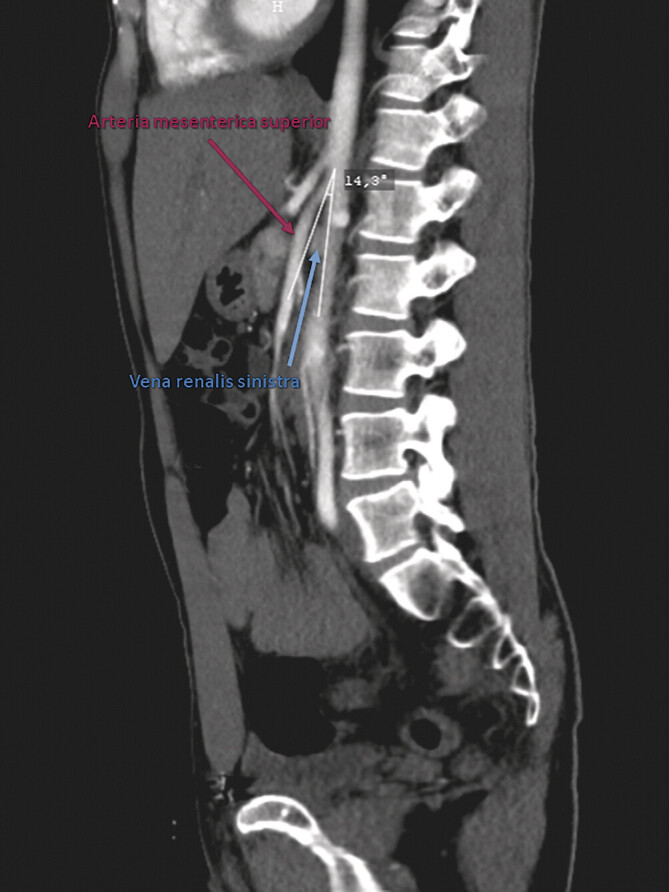
Angio-CT-Scan (sagittale Rekonstruktion mit arterieller Kontrastmittelphase): Kompression der linken im Querschnitt abgebildeten Nierenvene zwischen der A. mesenterica superior (ventral) und der Aorta abdominalis (dorsal) bei signifikant reduziertem Aortomesenterialwinkel (14,3°).

Die Kompression der linken Nierenvene zwischen Aorta abdominalis und A. mesenterica superior setzt einen verkleinerten aortomesenterialen Winkel ( Abb. 2 ) voraus, der im Regelfall zwischen 45° und 90° beträgt 7 8 . Es hat sich ein Winkel < 35–39° zur Diagnosestellung eines Nussknacker-Syndroms etabliert 9 , ein kleinerer Winkel ist hierbei mit einer höheren diagnostischen Genauigkeit assoziiert. Kim et al. zeigten bei einem Winkel < 39° in der Sagittalebene im CT eine Sensitivität von 92% und eine Spezifität von 89% 10 .

Für einen verkleinerten aortomesenterialen Winkel wird u. a. ein Mangel an mesenterialem Fettgewebe bei einem zumeist asthenischen Patientenhabitus verantwortlich gemacht und eine direkte Korrelation mit dem BMI nachgewiesen 11 12 . Bestätigt wird dieses pathophysiologische Modell durch eine Linderung der Beschwerdesymptomatik infolge einer Gewichtszunahme 13 . Auch durch eine Hyperlordose der LWS kann die Aorta abdominalis „angehoben“ und hierdurch der aortomesenteriale Winkel verkleinert werden 5 . Durch einen schlechten Ernährungsstatus und einen Mangel an stützendem retroperitonealem Fettgewebe kann eine Dorsalverlagerung der Niere zu einem Aufspannen der linken Nierenvene über der Aorta abdominalis zu einer Aggravierung der Beschwerden beitragen 14 .

**Abb. 3:**
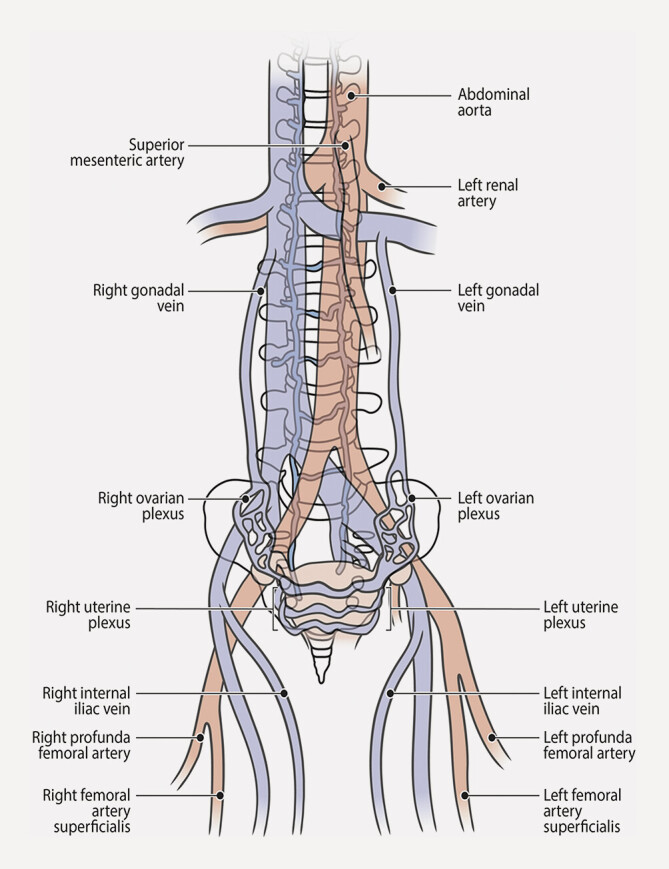
Schematische anatomische Darstellung der venösen Drainagewege bei Hochdruck der linken Nierenvene. Quelle: Image- & Bilderfundus des Arbeitsbereiches Gefäßchirurgie, Klinik für Allgemein-, Viszeral-, Gefäß- und Transplantationschirurgie Uniklinik Magdeburg.

Die Kompression der linken Nierenvene führt zu einem linksseitigen Nierenvenenhochdruck. Entsprechend Grimm et al. sind die wichtigsten Kollateralbahnen, die linke Gonadenvene und die kommunizierende Lendenvene ( Abb. 3 ), bei 16% bzw. 24% der NCS-Patienten dilatiert 15 . Findet sich eine suffiziente Drainage in die untere Hohlvene, ist das Krankheitsbild kompensiert und klinisch inapparent, man spricht hier vom Nussknacker-Phänomen, das mit einer Häufigkeit zwischen 8 und 30,5% beobachtet wird 16 . Bei einer insuffizienten Drainage kann die venöse Druckbelastung zu uncharakteristischen Beschwerden führen. So kann der retrograde Fluss über die linke 2. Lendenvene eine regionale Stauung des epiduralen Venenplexus bedingen, die dann zu erhöhtem Hirndruck und rezidivierendem täglichen Kopfschmerz führt 17 . Über die retrograd perfundierte Ovarial- bzw. Testikularvene füllt sich der pelvine Venenplexus, was das klinische Bild eines Becken-Stauungs-Syndroms, engl. „Pelvic Congestion Syndrome“, auslösen kann. Die retrograde Gonadenvenenperfusion wird durch den Umstand erleichtert, dass diese in 15% der Fälle keine Klappe aufweist 18 . Eine Inkompetenz der gonadalen Venenklappen wird in 40% der Fälle beschrieben 16 . Das Krankheitsbild „Pelvic Congestion Syndrome“ – 1949 durch Taylor geprägt 19 – wird durch einen pathologischen Reflux über Gonaden-, Gesäß- oder bzw. und Parauterinvenen hervorgerufen und hat eine Prävalenz von 15–43% 20 . Es ist assoziiert mit unspezifischen Beschwerden wie Dysmenorrhö (86%), Dyspareunie/postkoitale Schmerzen (40,8%), Unterleibschmerzen, Schmerzen bei Miktion/Defäkation, aber auch Varikozelen, Becken-, Vulva- (45,9%), Gluteal- oder Beinvarizen (58,7%) 21 . Das Nussknacker-Syndrom stellt neben insuffizienten Gonaden- und Iliakalvenen nur einen pathophysiologischen Auslöser der Erkrankung dar, eine derartige Symptomkonstellation sollte jedoch stets auch nach einem Nussknacker-Syndrom fahnden lassen. Der unmittelbare Nierenvenenhochdruck führt zu Stauungsschmerzen in der linken Lendengegend, zu einer Makrohämaturie durch Ruptur des dünnwandigen Septums zwischen den kleinen Venen und dem Sammelsystem des Nierenfornix 22 und zu einer orthostatischen Proteinurie 23 .

### Diagnostik

In Anbetracht des bunten Bildes aus unspezifischer pelviner, lumbaler oder auch kephalischer Schmerzsymptomatik und einer ggf. in der klinischen Untersuchung in Erscheinung tretender Varikose gluteal, an der Vulva, den Testes oder am Oberschenkel kommt der differenzialdiagnostischen klinischen Beurteilung große Bedeutung zu. Möglicherweise berichtet der Patient auch über eine Makrohämaturie.

Weiter erhärten lässt sich der Verdacht durch eine Urinuntersuchung mit Nachweis einer Hämaturie oder Proteinurie.

Der nächste diagnostische Schritt besteht in der noninvasiven Duplexsonografie, in der Kollateralvenen um die linke Nierenvene oder Beckenvarizen zu detektieren sind 24 . Weitere Hinweise gibt das Verhältnis des Innendurchmessers der linken Nierenvene am Nierenhilus zum Durchmesser am Kompressionspunkt an der A. mesenterica superior von > 3 in Rückenlagerung und > 5 in stehender Position 25 .

Durch Messung der Strömungsgeschwindigkeiten können nach dem Bernoulli-Prinzip der Kontinuität für ideale und inkompressible Flüssigkeiten die Druckgradienten zwischen V. cava inferior und der linken Nierenvene abgeschätzt werden. Die relevante Gleichung lautet:

P1 + ½ (ρv1 ^2^ ) + ρgh1 = P2 + ½ (ρv2 ^2^ ) + ρgh ^2^

wobei P der Druck, v die Geschwindigkeit, h die Höhe, ρ die Dichte des Blutes von etwa 1,06 g/cm ^3^ und g die Erdbeschleunigung ist. Eingesetzt und vereinfacht, entsteht die Formel:

Da bei gesunden Menschen kein wesentlicher Druckgradient zwischen V. cava inferior und der linken Nierenvene vorliegt 26 , kann bei einem Druckgradienten von > 3 mmHg die Diagnose eines Nussknacker-Phänomens bzw. -Syndroms gestellt werden 5 .

Der gleichen Arbeitsgruppe um Kim et al. gelang es, durch Messung der Spitzengeschwindigkeiten (PV – Peak Velocity) im Einmündungsbereich in die V. cava inferior und im hilären Bereich der V. renalis sinistra bei einem Quotienten von 5 die Diagnose eines Nussknacker-Syndroms mit einer Sensitivität und Spezifität von 80% bzw. 94% zu stellen 27 .

Malgor et al. konnten eine Korrelation von erweiterten parauterinen Venen > 5 mm sowie Ovarialvenen > 8 mm, insbesondere beim Vorhandensein eines pathologischen Refluxes während des Valsalva-Manövers für das Pelvic Congestion Syndrome nachweisen 28 .

Eine direkte Druckmessung ist durch invasive Katheterisierung möglich und gilt bislang noch als Goldstandard für die Diagnosestellung der Erkrankung 29 . Ein Druckgradient von > 3 mmHg zwischen linker Nierenvene und V. cava inferior sichert die Erkrankung 30 , während in der Normalpopulation selbiger < 1 mmHg beträgt 22 .

**Abb. 4:**
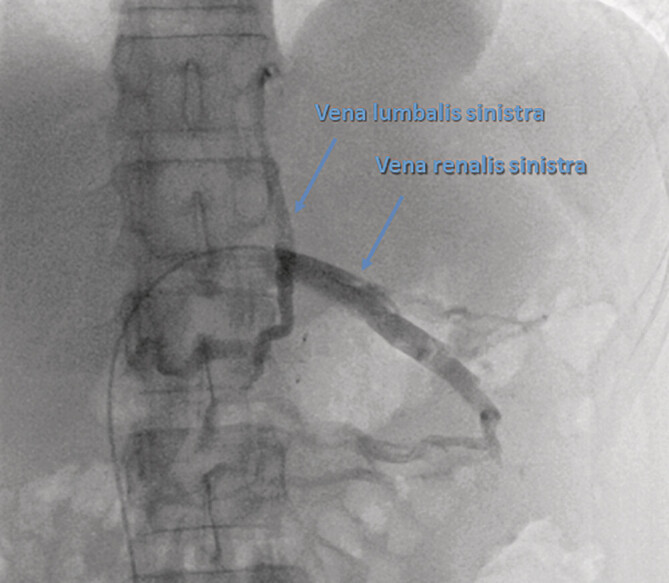
Digitale Subtraktionsangiografiesequenz: betonte, durchmessererweiterte V. lumbalis sinistra als proximale Kollaterale bei Abflussstörung der V. renalis sinistra infolge einer einmündungsnahen Kompression in die V. cava inferior bei bereits radiologisch interventionell suffizient verschlossener linker Gonadenvene.

Weiterhin kann die venöse Angiografie auch genutzt werden, um den retrograden Fluss in der linken Gonadenvene oder der linken 2. Lendenvene ( Abb. 4 ) nachzuweisen und hier auch in „gleicher Sitzung“ therapeutisch aktiv zu werden.

Das Angio-CT ist eine weit verbreitete Untersuchungsmodalität, die zur weiteren Abklärung einer unspezifischen abdominellen oder lumbalen Schmerzsymptomatik oder auch bei Makrohämaturie zum Einsatz kommt.

**Abb. 5:**
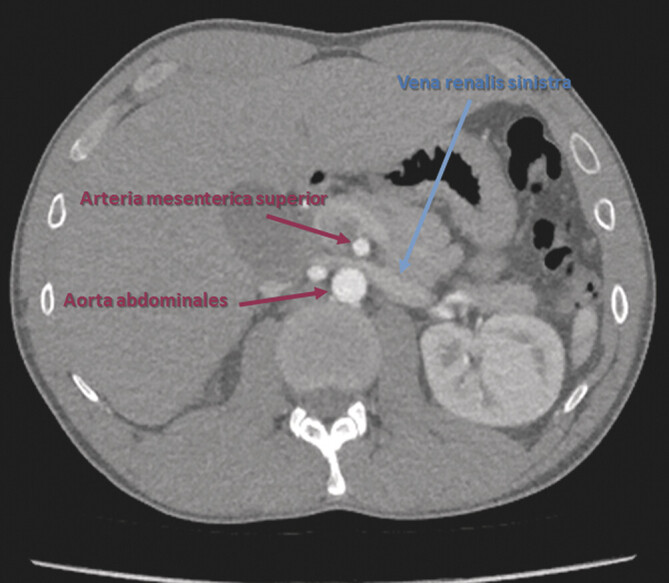
Angio-CT-Scan (axiale Rekonstruktion mit arterieller Kontrastmittelphase): „Schnabelzeichen“ – als schnabelartige Einengung der V. renalis sinistra zwischen A. mesenterica superior und Aorta abdominalis.

Charakteristisch ist hier der verkleinerte aortomesenteriale Winkel ( Abb. 5 ), der < 39° eine Sensitivität von 92% und eine Spezifität von 89% für die Diagnose eines Nussknacker-Syndroms hat 10 . Die Kompression der linken Nierenvene zwischen Aorta abdominalis und A. mesenterica superior stellt sich CT-morphologisch als sog. „Schnabelzeichen“ dar, das mit einer Sensitivität von 91,7% und einer Spezifität von 88,9% ebenfalls eine hohe diagnostische Genauigkeit zur Diagnose des Krankheitsbildes aufweist 31 . Von Kim et al. wurde das Kontrastmittel-Jetting-Phänomen des kontrastmittelärmeren venösen Flusses über die Stenose der linken Nierenvene in die V. cava inferior in der frühen kortikalen Phase als weiteres diagnostisches Zeichen gewertet, während sich in der Normalpopulation der venöse Abstrom eher als Massenbewegung darstellt 27 .

Die zur Abklärung der Hämaturie zum Einsatz kommende Zystoskopie kann in der Blutungsphase eine Blutungslokalisation aus dem linken Harnleiter sowie als indirektes Zeichen des Nussknacker-Syndroms Impressionen durch die Varizenstränge am Nierenbecken und Harnleiter nachweisen 32 .

**Abb. 6:**
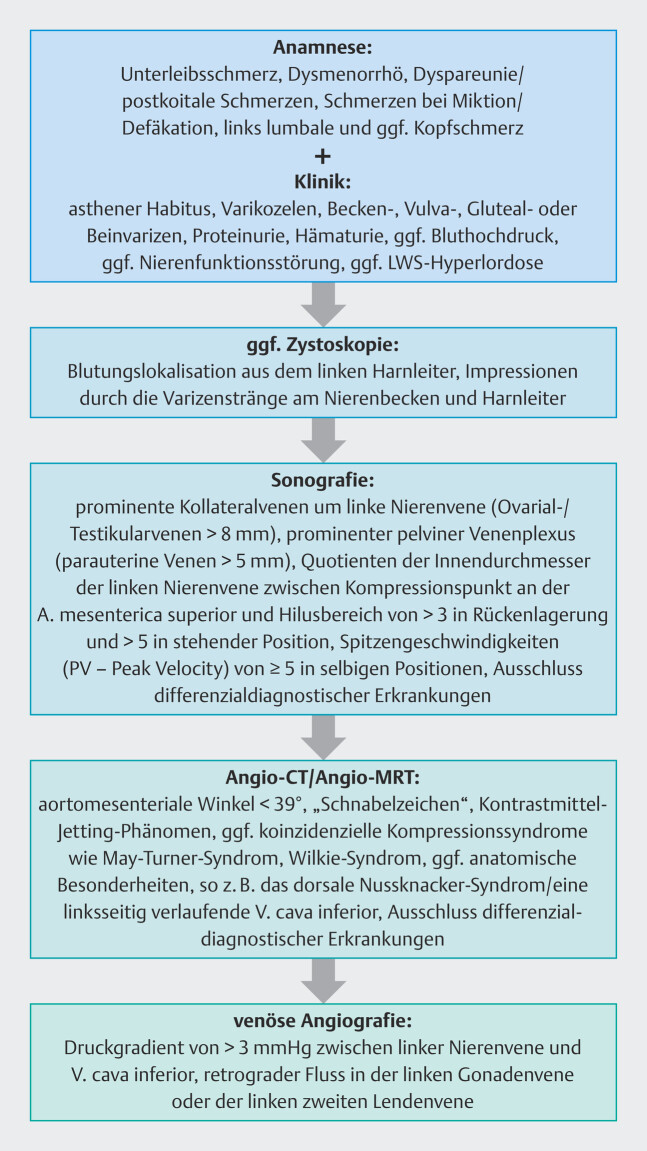
Diagnostischer Algorithmus – Nussknacker-Syndrom.

Unter Berücksichtigung der eruierbaren Expertise in der Literatur empfehlen wir den diagnostischen Algorithmus in Abb. 6 5 6 8 .

### Therapieoptionen

**Abb. 7:**
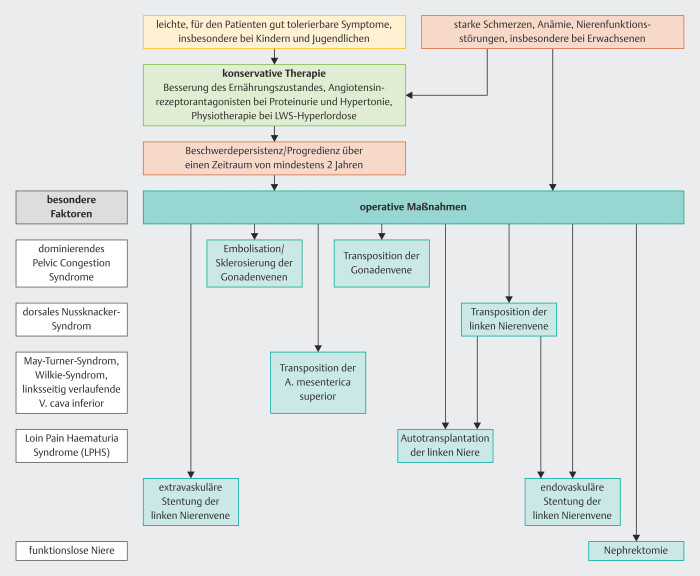
Therapieoptionen des Nussknacker-Syndroms.

Bei der Therapie des Nussknacker-Syndroms sind alle Optionen vertreten, die die moderne Medizin zu bieten hat, beginnend von konservativen diätetischen, medikamentösen, physiotherapeutischen Maßnahmen über radiologische Interventionen bis hin zu laparoskopischen und offen chirurgischen Verfahren ( Abb. 7 ). Die Auswahl des Verfahrens richtet sich zum einen nach den Beschwerden des Patienten, zum anderen nach den Erfahrungen und Strukturen der Versorgungseinrichtung.

Allein die Verlaufskontrolle mit Ausschluss eines komplikativen Verlaufs kann durch Reifung der venösen Kollateralisierung zu einer Druckregredienz in der linken Nierenvene und Remission der Beschwerden führen 5 .

Bei Kindern und Jugendlichen, insbesondere mit asthenischem Habitus, bei denen mit einer Zunahme des mesenterialen Fettgewebes und somit einem Aufrichten des aortomesenterialen Winkels mit Entstauung der linken Nierenvene gerechnet werden kann, sollte konservativ verfahren werden. Hier ist eine Ernährungstherapie der Patienten und deren Eltern indiziert. Selbiges Verfahren ist selbstverständlich auch bei entsprechend mangelernährter erwachsener Patientenklientel angezeigt. In einem Nachbeobachtungszeitraum von 26 Monaten während einer konservativen Behandlung mit Schwerpunkt auf Gewichtszunahme und einer Zunahme des retroperitonealen Fettgewebes konnten Scultetus et al. eine Symptomverringerung bei 30% der Patienten nachweisen 33 .

Bei einer Hyperlordose der Lendenwirbelsäule (LWS) kommen Rückenschule und physiotherapeutische Maßnahmen in Betracht, die neben dem Ziel einer Beschwerdelinderung der links-lumbalen und -pelvinen Schmerzsymptomatik des Nussknacker-Syndroms auch in einer Linderung bzw. Ausschaltung der chronischen LWS-Beschwerden und vertebrogener Schäden durch Fehlbelastung besteht.

Die orthostatische Proteinurie tritt, wie aktuellere Studien belegen konnten, während der Adoleszenz mit einer Inzidenz von > 15% auf 34 . Wie auch für das Nussknacker-Syndrom konnten ein schlanker Habitus und eine Hyperlordose als Risikofaktoren herausgearbeitet werden 35 , in ⅔ der Fälle lässt sich sogar eine Kompression der linken Nierenvene als Hinweis auf ein Nussknacker-Syndrom nachweisen 36 . Pathophysiologisch werden glomeruläre Läsionen und eine Überaktivierung des Noradrenalin- und Angiotensin-II-Stoffwechselsystems vermutet 35 . Eine venöse Druckerhöhung scheint in der Genese der Proteinurie eine Schlüsselrolle zu spielen, wie zahlreiche Fallberichte, bspw. bei einer Patientin mit Nierenvenenkompression durch eine große Milzzyste oder in einem weiteren Fall durch ein extremes Kinking der Nierenvene mit Normalisierung nach entsprechender Therapie belegen 37 38 . Für den Einsatz der ACE-Hemmer zur Hemmung des überaktivierten Angiotensin-II-Stoffwechsels ist neben einer Therapie der Proteinurie auch eine Besserung der ggf. begleitenden arteriellen Hypertonie belegt 39 .

### Sklerosierung/Embolisation bei symptomatischen Ovarial- bzw. Testikularvarizen

Bei Patienten mit Fokus auf einer pelvinen Beschwerdesymptomatik wird von verschiedenen Autoren die primäre Embolisation der linken Gonadenvene empfohlen 40 41 . Seit der Einführung der radiologisch interventionellen bilateralen Embolisation der Ovarialvenen durch Edwards et al. 1993 beim Pelvic Congestion Syndrome 42 hat selbiges Verfahren breite Akzeptanz für dieses Krankheitsbild gewonnen und führt in etwa 75% zur Beschwerdefreiheit der Patientinnen 43 . Aufgrund der multifaktoriellen Genese des Pelvic Congestion Syndrome ist es unabdingbar, fortwährend differenzialdiagnostische Überlegungen in das Therapiekonzept einfließen zu lassen. So wird von Rastogi et al. in ihrem Fallbericht bei einer 32-jährigen Patientin und langjährig persistierenden Beschwerden nach Beckenvenenstentung bei einem May-Turner-Syndrom auf die Abklärung hinsichtlich einer Kompetenz der beiden Gonadenvenen und des Vorliegens eines Nussknacker-Syndroms bei simultan vorliegendem Pelvic Congestion Syndrome hingewiesen 44 .

Die Okklusion der Gonadenvenen kann über die V. femoralis, V. cephalica, V. basilica oder V. jugularis mittels Coil-Embolisation oder Sklerosierung mittels Polidocanol und Natriumtetradecylsulfat durchgeführt werden 45 . Unspezifische entzündliche Schmerzen können mit NSAR behandelt werden, Komplikationen sind Lungenarterienembolien durch Dislokation des Embolisates, Venenperforationen und tiefe Venenthrombosen 45 .

### Endovaskuläre Stentung der linken Nierenvene

Der erste endovaskuläre Stent für NCS wurde 1996 beschrieben 46 . Hauptproblem bei der Nierenvenenstentung ist die Stentdislokation von 6,7% über V. cava inferior in das rechte Herz, insbesondere bei Kindern und Jugendlichen mit noch nicht abgeschlossener Wachstumsphase 47 . Um diese Komplikation zu verhindern, muss der Stent ausreichend dimensioniert sein. Meistenteils kommen Stents mit einem Durchmesser von 12–16 mm und 60–80 mm in der Länge zum Einsatz. Ein „Oversizing“ des Stents scheint mit einer erhöhten postinterventionellen Schmerzsensation zu korrelieren 48 . Der Stent sollte proximal etwa 3–5 mm in die V. cava inferior (IVC) reichen und distal die linke Ovarialveneneinmündung überragen, ohne die Segmentvenen zu affektieren. Nach der Stentplatzierung sollte erneut der Druckgradient zwischen der IVC und der distalen Nierenvene gemessen werden, wobei bei einem Gradienten > 2 mmHg eine Nachdilatation erforderlich ist. Die Intervention kann im ambulanten Setting erbracht werden und die Patienten mit einer dualen Thrombozytenaggregationshemmung, bestehend aus Aspirin und Clopidogrel für 1–3 Monate, in die Häuslichkeit entlassen werden 41 . Die Offenheitsrate nach 2 Jahren beträgt 85,2% 49 .

### Transposition der linken Nierenvene

Die Transposition der linken Nierenvene gilt derzeit als chirurgisches Standardverfahren bei unter konservativer Therapie persistierenden Beschwerden 50 . Die linke Nierenvene wird im Bereich der Einmündung in die V. cava inferior abgesetzt und weiter distal spannungsfrei in End-zu-Seit-Technik in die V. cava inferior re-inseriert. Alternativ ist auch die End-zu-Seit-Anastomose in die linke Beckenvene möglich. Bei unzureichender Venenlänge, u. a. auch bei einer Einkürzung aufgrund einer Druckschädigung der Venenwand, kann eine Interposition mittels V. saphena magna erfolgen. Die anteriore Transposition der linken Nierenvene stellt auch das Verfahren der Wahl bei einem posterioren Nussknacker-Syndrom dar 51 . Das offen chirurgische Vorgehen wird zunehmend durch minimalinvasive laparoskopische oder auch roboterassistierte Verfahren abgelöst 52 . Erben et al. haben ihr Patientenkollektiv über einen 20-Jahres-Zeitraum von 1994 bis 2004 untersucht. Obwohl 87% aller Patienten nach der OP beschwerdefrei waren, mussten 8% innerhalb der ersten 30 Tage und 22% nach 30 Tagen, zumeist auf dem Boden einer Stenosesymptomatik, in einem Fall aufgrund eines Gefäßverschlusses, zumeist in Form einer Stentimplantation, revidiert werden. Die Re-Interventionsrate nach 1 Jahr betrug 68% und nach 2 Jahren 74% 53 .

### Autotransplantation der linken Niere

Die Autotransplantation ist neben dem endovaskulären Stenting der linken Nierenvene eine Salvage-Strategie nach frustraner Transposition der linken Nierenvene. Insbesondere beim simultan diagnostizierten Lendenschmerz-Hämaturie-Syndrom (Loin Pain Haematuria Syndrome, LPHS), das auch mit dem Nussknacker-Syndrom in Verbindung stehen kann, wird infolge einer Denervierung bei der Nierentransplantation in linke oder rechte Beckengrube eine Schmerzausschaltung erreicht 54 . Systemimmanent ist selbstverständlich das erhöhte Komplikationspotenzial durch die 3 Anastomosen von Nierenvene, -arterie und Harnleiter.

### Transposition der Gonadenvene

Die Transposition der linken Gonadenvene in die V. cava inferior oder optional in die linke Beckenvene kann sowohl in offen chirurgischer als auch minimalinvasiver Technik zur Behandlung eines Pelvic Congestion Syndrome im Rahmen eines Nussknacker-Syndroms zum Einsatz kommen. Die Arbeitsgruppe um Gilmore et al. konnte in der eigenen Patientenklientel eine Beschwerderemission in 61,1% und eine Beschwerderegression in 22,2% der Fälle ohne krankheitsspezifische Letalität oder Notwendigkeit einer Revision belegen 55 .

### Extravaskuläres Stenting der linken Nierenvene

Mithilfe einer extravaskulären Stentplatzierung kann völlig auf eine Gefäßreanastomosierung mit spezifischen Komplikationen oder auf das Ausklemmen der Niere verzichtet werden. Dieses Verfahren wurde 2010 erstmals durch Zhang et al. in einer Fallserie von 3 Patienten beschrieben 56 . Die Ergebnisse sind vielversprechend. So konnten Steinberg et al. in einer Fallserie während eines 3-Jahres-Zeitraumes von 2016 bis 2019 an 6 Patienten im Alter von 45 ± 6 Jahren bei allen Patienten eine sofortige Schmerzlinderung und in 50% eine Besserung der weiteren Symptome erheben. Die Operation wurde – robotisch assistiert – mit einer Operationsdauer von 143 ± 20 min durchgeführt. Verwendet wurde ein Polytetrafluorethylen-Gefäß-Stent mit einer Länge von 2,25 ± 0,3 cm mit einem Durchmesser von 1 cm, die beginnend von der linken Nebennierenvene bis zur Einmündung in die V. cava inferior platziert wurden 57 58 .

Eine Weiterentwicklung, die den anatomischen Gegebenheiten gerecht wird und auch eine Lösung für die Patientenklientel in der Wachstumsphase sein könnte, stellt der 3-dimensional gedruckte Polyetheretherketon-Stent (PEEK-Stent) dar 59 .

### Nephrektomie

Die Nephrektomie wird lediglich bei Funktionslosigkeit der linken Niere als Ultima Ratio durchgeführt.

### Transposition der A. mesentica superior

**Abb. 8:**
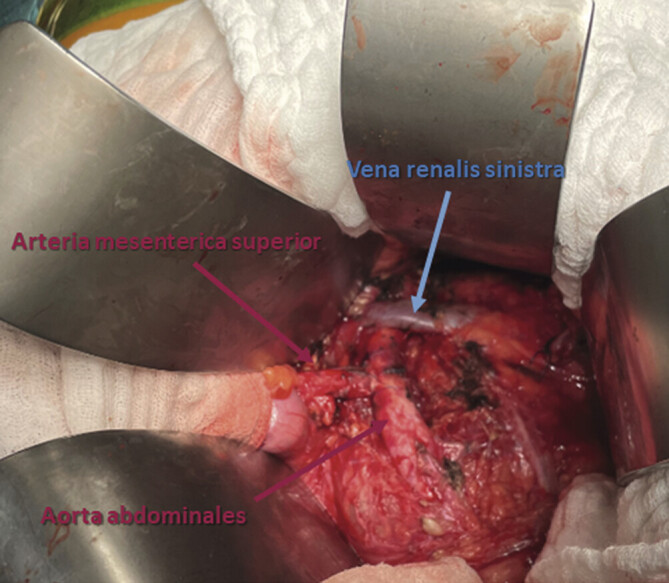
Intraoperativer Situs mit distal in die Aorta abdominalis re-inserierter A. mesenterica superior und nun zwar noch chronisch dilatierter, aber dekomprimierter V. renalis sinistra.

**Abb. 9:**
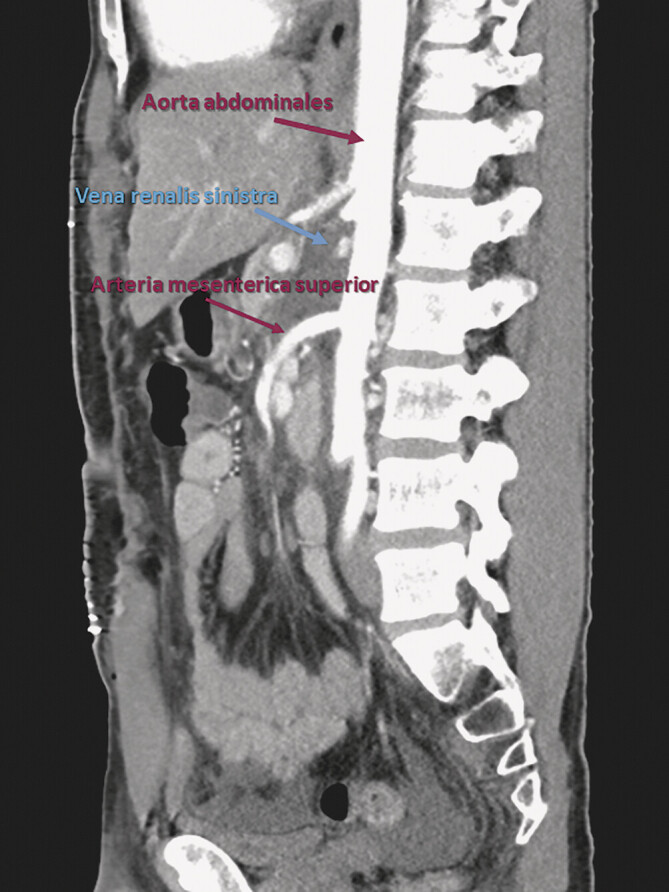
CT des Abdomens – sagittale Rekonstruktion mit arterieller Kontrastmittelphase: Nach distal auf die Aorta abdominalis transponierte A. mesenterica superior mit nun dekomprimierter V. renalis sinistra.

Die Transposition der A. mesenterica superior ( Abb. 8 , Abb. 9 ) nach distal auf der infrarenalen Aorta abdominalis ist eine kausale Behandlungsoption für das Nussknacker-Syndrom, die trotz Nachweis von Effektivität und Sicherheit, möglicherweise aufgrund des Risikos einer mesenterialen Ischämie, eine eher untergeordnete Rolle spielt. In der PubMed-Literaturrecherche werden lediglich 7 Einträge gefunden. In der Arbeit von Zhang et al. wurden 3 von 20 Patienten mit einer Transposition der Mesenterialarterie, 2 mit einer Transposition der linken Nierenvene und 15 mit einem endovaskulären Stent der linken Nierenvene behandelt. Einer der 3 Patienten mit einer Mesenterialarterientransposition musste aufgrund eines lokalen Hämatoms revidiert werden, bei einem mittels Nierenvenenstent behandelten Patienten kam es zu einer Stentdislokation. Alle Patienten haben hinsichtlich ihrer Symptombehandlung von den operativen Maßnahmen profitiert 9 . In einer kleineren Fallserie von Lin et al. aus 2003 erhielten 3 Patienten eine Mesenterialarterientransposition und 3 Patienten eine endovaskuläre Nierenvenenstentung. In der Gruppe der Transpositionen wurde eine revisionspflichtige Blutung und ein paralytischer Ileus dokumentiert, in der Gruppe der stentversorgten Patienten eine Revision wegen Stentdislokation. Von den Autoren wurde die Stentversorgung als die risikoärmere Behandlungsmodalität gewertet 60 . Ali et al. untersuchten 10 Patienten mit simultan bestehendem Nussknacker- und Wilkie-Syndrom, einer Kompression der Pars horizontalis des Duodenums zwischen Aorta abdominalis und A. mesenterica superior. Die Erfolgsrate war 100%, in keinem der Fälle traten Blutungen oder eine Mesenterialischämie auf, ein Patient wurde nach 2 Jahren aufgrund einer 60% proximalen A.-mesenterica-superior-Stenose mit einem Stent behandelt 61 . Ein Fallbericht aus Budapest/Ungarn konnte ebenfalls einen komplikationslosen erfolgreichen Verlauf darstellen 62 . Die Gruppe um Yang et al. referierten 2012 über einen 20-jährigen Mann mit einer schweren Hämaturie, der aufgrund einer Kompression der linken Nierenvene und einer linksseitigen V. cava inferior über einen links-paramedianen retroperitonealen Zugang mittels Transposition der A. mesenterica superior erfolgreich und ohne Komplikationen versorgt wurde 63 .

## Repräsentativer Fallbericht

**Abb. 10:**
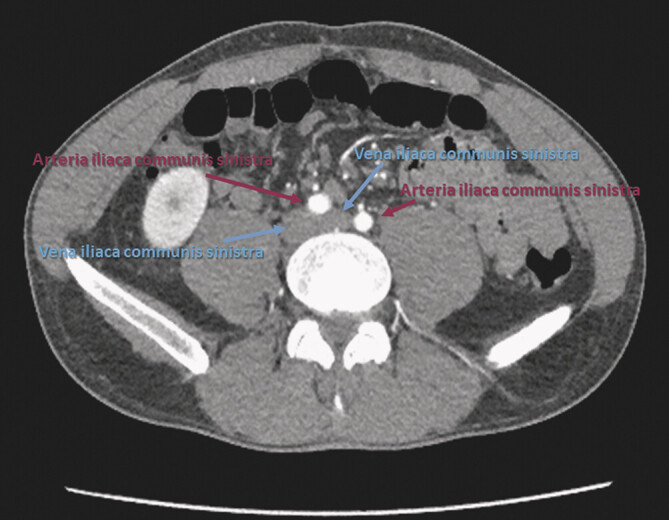
CT des Abdomens – Transversalscan (axiale Rekonstruktion mit arterieller Kontrastmittelphase): May-Turner-Syndrom infolge der Kompression der Beckenvenen durch die Beckenarterien auf dem Lendenwirbelkörper mit Ausbildung eines Beckenvenensporns.

Ein 32-jähriger Patient stellte sich aufgrund einer episodischen, seit 2 Jahren bestehenden Schmerzsymptomatik in der linken Flanke, linken Leiste und Skrotum vor. Die Schmerzen würden beim Koitus aggravieren. Gelegentlich würden migräneartige Kopfschmerzen auftreten. Anamnestisch konnten ein Heuschnupfen, ein Zustand nach Tonsillektomie, eine Pexie bei rechtsseitigem Pendelhoden und eine Reposition bei Nasenbeinfraktur eruiert werden. In der klinischen Untersuchung stellt sich ein asthenischer Habitus mit einem BMI von 18 kg/m ^2^ ohne weitere klinische Auffälligkeiten dar. Der Patient war gerade aufgrund der chronischen Schmerzsymptomatik als Soldat auf Zeit ausgemustert worden. Er berichtete, seit etwa 5 Jahren etwa 1-mal pro Jahr antibiotisch an einer linksseitigen Epididymitis behandelt zu werden. In der Urinuntersuchung war eine Proteinurie ohne Hämaturie nachgewiesen worden. Über die hiesige radiologische Klinik wurde dann eine urologisch vordiagnostizierte linksseitige Varikozele mittels distaler Coil-Embolisation (4 × 2/2 Hilal, 3 × 2/5 Tornado), Sklerosierung des Zwischensegmentes mit 3% Aethoxysklerol und proximaler Mikroplugembolisation (5 mm Amplatzer Vascular Plug 4, Abbott Cardiovascular, Plymouth, Großbritannien) der linken V. spermatica behandelt worden. Während der Untersuchung waren eine retrograd perfundierte linke Testikularvene und ausgeprägte Umgehungskreisläufe der linken Nierenvene beschrieben worden. Vier Wochen später waren die linkstestikulären Schmerzen wieder aufgetreten. Sonografisch stellten sich die linken Testikularvenen im Seitenvergleich mit 2 mm gering erweitert dar. Nach 6 Monaten war eine venöse Angiografie durchgeführt worden und hier neben einem vorderen Nussknacker-Syndrom mit einem pelvinen venösen Blutfluss von links nach rechts auch ein May-Turner-Syndrom mit retrogradem Fluss in der inneren Beckenvene unter Valsalva-Manöver beschrieben worden. Nach der stattgehabten Embolisationstherapie der linken V. testicularis zeigte diese keinen retrograden Fluss mehr, wohl aber selbigen über lumbale Venenkonvolute ( Abb. 4 ). Fünf Monate später war eine Angio-CT-Untersuchung ( Abb. 2 ) durchgeführt worden, in der sich ein verkleinerter Aortomesenterialwinkel von 15°, eine Kompression der linken V. renalis ( Abb. 5 ) auf Höhe der A. mesenterica superior (Schnabelzeichen) und eine Kompression der linken V. iliaca communis durch die rechte A. iliaca communis (May-Turner-Syndrom; Abb. 10 ) fanden. Als weiterhin auffällig wurde ein prominenter Plexus venosus prostaticus beschrieben. In Zusammenschau aller Befunde und dem Aspekt einer hohen Expertise in der offen chirurgischen Aortenchirurgie als zertifiziertes Aortenzentrum wurde dem Patienten eine Transposition der A. mesenterica superior empfohlen, der sich der Patient anschloss, woraufhin nach orthograder Darmvorbereitung mit Natriumpicosulfat, Thromboseprophylaxe mit niedermolekularem Heparin und „Single-Shot“-Antibiotikagabe mit Cefuroxim und Metronidazol die Operation durchgeführt wurde.

Hierbei wurde die linke Kolonflexur mobilisiert und die A. mesenterica superior nach Anschlingen der linken Nierenvene infrapankreatisch dargestellt. Nach systemischer Gabe von 5000 IE Heparin wurde die A. mesenterica superior abgangsnah mittels Perlon-Durchstichligatur der Stärke 1 abgesetzt und infrarenal nach Ausklemmung der Aorta abdominalis in selbige mit Prolene 5–0-Naht in Seit-zu-End-Technik re-inseriert ( Abb. 8 ). Die Ausklemmzeit der A. mesenterica superior betrug 22 min, die Schnitt-Naht Zeit 2 h und 56 min. Vonseiten der Anästhesie war ein Blutverlust von 100 ml bilanziert worden.

Der Patient wurde postoperativ auf der operativen Intensivmedizin weiterbetreut: Nach Gerinnungskontrolle wurde eine intravenöse Heparinisierung ohne Verlängerung der partiellen Thromboplastinzeit initiiert. Bereits am Operationsabend war keine Proteinurie mehr nachweisbar. Bei klinischem (schmerzfrei und kreislaufstabil) Wohlbefinden und paraklinisch unauffälliger Konstellation (normaler Laktatwert) sowie unauffälliger Sekretion über die Drainage im kleinen Becken konnte der Patient am 1. postoperativen Tag auf die gefäßchirurgische Normalstation zurückverlegt werden. Hier erhielt der Patient eine Frühmobilisation sowie den Kostaufbau mit Suppe am 2. postoperativen Tag. Die Drainage konnte ebenfalls am 2. postoperativen Tag entfernt werden. Aufgrund einer isolierten Leukozytose von 18 Gpt/l wurde bei subjektivem Wohlbefinden und reizlosen Wundverhältnissen eine Angio-CT durchgeführt mit Nachweis einer regelrechten Viszeralperfusion über die nun in nahezu rechtem Winkel in die Aorta abdominales re-inserierte A. mesenterica superior ( Abb. 9 ) ohne Zeichen einer Komplikation oder sonstigen Infektion. Auf eine antibiotische Therapie wurde daher verzichtet. Der weitere Kostaufbau wurde vom Patienten bei regelrechter Darmentleerung gut vertragen. Der Patient berichtete weder über Schmerzen in Flanke, Skrotum noch des Kopfes. Bei weiter abfallenden Entzündungsparametern und subjektivem Wohlbefinden war am 6. postoperativen Tag die Entlassung des Patienten unter ASS 1 × 100 mg p. o. in die Häuslichkeit möglich. Bei der planmäßigen ambulanten Wiedervorstellung nach 3 Monaten berichtete er über eine komplette Remission der initial bestandenen Schmerzsymptomatik, die Wundverhältnisse stellten sich reizlos dar.

## Diskussion

Das Nussknacker-Syndrom ist eine Erkrankung, die sich chamäleonartig hinter vielen unspezifischen Beschwerden wie dem linksseitigen Flankenschmerz, dem Kopfschmerz, Becken-, Defäkations-, Miktions- oder koitalen Schmerzen verbergen kann. Hämaturie oder Proteinurie sind wichtige diagnostische Hinweise auf die renovenale Hochdruckerkrankung. Weitere klinische Indizien können vor allem linksbetonte Beinvarizen, Varizen an Vulva, dem Gesäß oder Varikozelen sein. Mittels Duplexsonografie vom zumeist erstbehandelnden Gynäkologen oder Urologen können vor allem (die) linksseitig dominant-dilatierte Beckenvenen und Gonadenvene als Hinweis einer links-renovenalen Abflussbehinderung erhoben werden. In erfahrender Hand können duplexsonografisch Quotienten der Innendurchmesser der linken Nierenvene zwischen Kompressionspunkt an der A. mesenterica superior und Hilusbereich von > 3 in Rückenlagerung und > 5 in stehender Position 25 sowie der Spitzengeschwindigkeiten (PV – Peak Velocity) von ≥ 5 in selbigen Positionen die Diagnose eines Nussknacker-Syndroms mit einer Sensitivität und Spezifität von 80% bzw. 94% stellen 27 .

CT- oder MR-morphologisch lassen sich das „Schnabelzeichen“ und der verkleinerte Aortomesenterialwinkel < 39° mit hoher Sensitivität (91,7% bzw. 92%) und Spezifität (88,9% bzw. 89%) für das Krankheitsbild nachweisen 10 31 . Als diagnostischer Goldstandard gilt die invasive venöse Katheterisierung mit Bestimmung eines Druckgradienten von > 3 mmHg zwischen linker Nierenvene und V. cava inferior zur Sicherung der Erkrankung 30 .

Die Behandlungsoptionen des Nussknacker-Syndroms sind mannigfaltig und reichen von der reinen klinischen Kontrolle bis hin zur Nephrektomie. Entscheidend für die Auswahl des Behandlungskonzeptes sind Patientenalter, Habitus, Manifestation der klinischen Beschwerden unter Berücksichtigung der therapeutischen Anamnese. Koinzidenzielle Kompressionssyndrome, wie May-Turner-Syndrom, Wilkie-Syndrom, sollten ebenso wie anatomische Besonderheiten, so z. B. das dorsale Nussknacker-Syndrom, eine linksseitig verlaufende V. cava inferior oder das Lendenschmerz-Hämaturie-Syndrom (Loin Pain Haematuria Syndrome, LPHS) in die therapeutische Strategieplanung einbezogen werden.

Leichte, für den Patienten gut tolerierbare Symptome können konservativ verfolgt werden. Gerade bei unter 18-Jährigen tritt in 75% der Fälle infolge der weiteren körperlichen Entwicklung, Besserung des Ernährungszustandes mit Zunahme des mesenterialen stützenden Fettgewebes eine spontane Remission der Beschwerden ein 16 . In dieser Altersgruppe wird eine konservative Therapie über mindestens 2 Jahre angestrebt, bevor bei Beschwerdepersistenz eine Ausweitung der Therapie eingeleitet wird. Angiotensinrezeptorantagonisten können Proteinurie und Hypertonie gut behandeln 39 . Rezidivierende Makrohämaturie mit Ausbildung einer Anämie, von starken Flankenschmerzen, bereits eingetretenen Nierenfunktionsstörungen erfordern eine zügige Behandlung.

Insbesondere unter Berücksichtigung des dominierenden jungen Patientenalters stellt sich die Frage nach einer niedrig invasiven, eher symptomatischen Therapie wie der Gonadenvenenembolisation oder einer kausalen, jedoch deutlich invasiveren Behandlung in Form einer Transposition der linken Nierenvene oder der A. mesenterica superior. Im klinischen Alltag wird zumeist ein Stufenkonzept gewählt und bei entsprechender Unterleibssymptomatik zunächst eine Gonadenvenensklerosierung durchgeführt, was möglicherweise auch der unvollständigen Diagnosestellung eines Nussknacker-Syndroms zum Behandlungszeitpunkt zuzuschreiben ist. Es gibt lediglich Fallberichte, die diesen Algorithmus unterstreichen. So wählten auch Aghdasi et al. zunächst die Ovarialvenenembolisation bei einer 26-jährigen Frau mit linksseitiger Beinschwellung, Dysmenorrhö und lumbaler Schmerzsymptomatik, bevor sie bei anhaltender Beschwerdesymptomatik über 3 Monate eine Stentung der linken Nierenvene initiierten 41 . Interessant ist – wie im eigenen Fallbeispiel – das simultane Auftreten eines May-Turner-Syndroms, das vom pathophysiologischen Erklärungsmodell einer Kompensation durch venöse Druckentlastung erschwert.

In einer „Medline“-Recherche untersuchten Velasquez et al. den Zeitraum zwischen 10/82 und 07/17 und fanden 17 Referenzen, von denen 47% eine offen chirurgische Operation – zumeist Transposition der linken Nierenvene – nachrangig eine renale Autotransplantation erhielten. In 41% der Fälle wurde eine endovaskuläre Stentversorgung durchgeführt, in 11,7% der Fälle eine minimalinvasive extravaskuläre Stentimplantation. Das Team spekulierte, dass infolge einer Weiterentwicklung die endovaskuläre Therapie an Bedeutung gewinnen wird 64 .

Bei noch nicht abgeschlossener Wachstumsphase ist das endovaskuläre Stenting kritisch zu sehen, da hier eine Stentmigration zu befürchten ist. Das extravaskuläre Stenting, gerade mit einem konfektionierten 3-dimensional gedruckten PEEK-Stent ist eine vielversprechende neue Lösung, die zudem minimalinvasiv mit hoher intraoperativer Übersicht am OP-Roboter erbracht werden kann 59 .

## Fazit

Das Nussknacker-Syndrom als venöse Abflussbehinderung der Niere stellte, medizinisch gesehen, bislang selbst eine recht „harte Nuss“ dar, wofür die moderne Medizin durch Evidenzfindung nicht zuletzt durch Fallberichte und retrospektive Kohortenanalysen mittlerweile einen verifizierten diagnostischen Algorithmus bereithält und auch für die vorwiegend junge Patientenklientel mit häufig synchron bestehenden weiteren Gefäßanomalien eine Auswahl an hocheffektiven Therapiemaßnahmen zur Verfügung stehen.
